# Comparison of Antipsychotics in the Treatment of COVID-19-Induced First-Episode Psychosis: A Review of Case Studies

**DOI:** 10.7759/cureus.103021

**Published:** 2026-02-05

**Authors:** Gurraj Singh, Riley Hartnett, Briana M Silva, Sayed Mohammad M Fekrat, Sakshi Prasad, Gurtej Gill, Sasidhar Gunturu

**Affiliations:** 1 Psychiatry, Bergen New Bridge Medical Center, Paramus, USA; 2 Psychiatry, BronxCare Health System, New York, USA; 3 Psychiatry, Richmond University Medical Center, Staten Island, USA; 4 Psychiatry, Kabul University of Medical Sciences, Kabul, AFG

**Keywords:** antipsychotics, covid-19, first-episode psychosis, psychosis, sars-cov-2

## Abstract

This study aims to systematically review COVID-19-associated first-episode psychosis cases, comparing antipsychotic selection, dosing strategies, treatment response timelines, adverse effects, and relapse rates to inform evidence-based pharmacological management.

We conducted a structured narrative review of published case reports and series describing COVID-19-Induced first-episode psychosis treated with antipsychotics. A comprehensive search of PubMed and Google Scholar (Jan 2020-Apr 2023) identified 42 eligible cases based on predefined inclusion/exclusion criteria. Data were extracted using a standardized template and summarized descriptively due to clinical heterogeneity. Variables included demographics, psychiatric features, antipsychotic(s) used, clinical course, and outcomes.

First-episode psychosis (FEP) was higher in males (24, 57.1%) and the 30-39 age group (10, 23.8%). Olanzapine was the most commonly used single antipsychotic (6, 28.6%), while the combination of haloperidol and aripiprazole was the most frequently used antipsychotic regimen (4, 19.0%).

Atypical antipsychotics were preferred (54.8%), with olanzapine (23, 54.8%) being the most commonly used at a mean dose of 10.9 mg/day. Reported side effects included fatigue, weight gain, akathisia, leukocytosis, and QT-interval prolongation (5, 11.9%), with a relapse rate of (2, 4.8%).

This review evaluates the treatment methods for COVID-19 FEP and develops a deeper understanding of various antipsychotics used in managing psychosis and its outcomes.

## Introduction and background

In 2020, the World Health Organization (WHO) declared COVID-19, caused by SARS-CoV-2, a global pandemic [1]. Since 2020, the COVID-19 pandemic has underscored numerous detrimental effects on the global health system. These include profound economic and medical impacts that extend beyond the purely physical realm and have consequences for mental health [2,3]. COVID-19 can cause a wide range of manifestations, from asymptomatic to mild respiratory symptoms and even fatal severe acute respiratory syndrome [4]. However, in rare cases, it has also been associated with neuropsychiatric manifestations, including psychotic features, particularly in those with no prior psychiatric history. Additional neuropsychiatric manifestations can also include, but are not limited to, delirium, encephalopathy, seizures, vertigo, hyposmia/anosmia, and consciousness disorders [5].

While case studies involving psychiatric symptoms associated with COVID-19 are rare and, consequently, constrained by small sample sizes, they consistently reveal similar findings. Instances of new-onset severe anxiety, agitation, paranoia, and disorganized thinking were frequently observed. Notably, none of the typical COVID-related respiratory or gastrointestinal symptoms, such as disturbances in taste and smell, were reported [4].

The precise mechanisms by which the coronavirus affects the brain remain unknown. According to current literature, COVID-19 is neurotropic and can directly infect the central nervous system (CNS) through various mechanisms. One elucidated pathway involves the olfactory neural pathway and brain capillary endothelial cells, facilitated by transmembrane angiotensin-converting enzyme 2 (ACE-2) receptors. Additionally, the literature indicates that the coronavirus is linked to the emergence of new psychosis resulting from a pronounced inflammatory response, thereby contributing to the psychotic symptoms observed in affected individuals [4,6]. It has been postulated that the profound inflammatory response to COVID-19 infection, known as the *cytokine storm*, produces neuropsychiatric symptoms through immunological mechanisms [7,8]. COVID-19 encephalopathy is hypothesized to be the direct result of cerebrovascular damage, secondary to the procoagulant status, or the indirect result of hypoxia from either the immune response or the medication [9]. Other studies proposed that the neuropsychiatric manifestations of COVID-19 may be due to post-infectious neuronal autoimmunity [10].

Although the relationship and neurogenesis between new-onset psychosis associated with COVID-19 infection warrant further investigation, treatment of the psychotic symptoms with antipsychotic medication, based upon current literature, improves or resolves these neuropsychiatric symptoms with complete recovery. As noted in Chaudhary et al., 41 (72%) patients responded to a low-to-moderate dose of antipsychotic with a full and quick recovery time [11,12]. The use of antipsychotics with appropriate medical intervention alongside early detection can shorten the duration of psychotic episodes and mitigate potential long-term neuropsychiatric consequences.

This study aims to systematically review antipsychotic treatment strategies for COVID-19-induced first-episode psychosis by comparing medication selection, clinical outcomes, and treatment responses to inform evidence-based management of this emerging psychiatric complication.

Methodology

Study Design

This study was conducted as a structured narrative review of published case reports and case series describing first-episode psychosis (FEP) associated with COVID-19 and treated with antipsychotic medications. Given the rarity of COVID-19-associated FEP and the predominance of case-based evidence, a descriptive synthesis approach was selected rather than a systematic review with meta-analysis. Because of the descriptive narrative design and the nature of the included literature, a Preferred Reporting Items for Systematic Reviews and Meta-Analyses (PRISMA) flow diagram was not generated.

Data Sources and Search Strategy

A comprehensive literature search was performed using PubMed (National Library of Medicine) and Google Scholar [4,13-46]. The search included articles published between January 2020 and April 2023, corresponding to the onset and progression of the COVID-19 pandemic.

The search strategy employed combinations of keywords related to COVID-19 (e.g., “COVID-19” or “SARS-CoV-2”), psychosis (e.g., “psychosis,” “FEP,” or “new-onset psychosis”), and treatment or clinical context (e.g., “antipsychotic” or “neuropsychiatric manifestations”). In addition, reference lists of eligible articles were manually reviewed to identify further relevant studies. Only articles published in English were included.

Eligibility Criteria

Inclusion criteria: Eligible studies were case reports or case series describing new-onset psychosis or FEP in which psychotic symptoms occurred during or following confirmed COVID-19 infection. To ensure cases reflected new-onset illness, included patients had no documented prior psychiatric history, including psychotic, mood, or substance use disorders. Antipsychotic medication also needed to be used as a primary component of psychosis management. Patients of any age, sex, or geographic location were eligible.

Exclusion criteria: Studies were excluded if there was any prior psychiatric diagnosis, regardless of severity. Cases were also excluded when psychotic symptoms were attributable primarily to substance intoxication or withdrawal, or when antipsychotics were not used or were not central to clinical management.

Study Selection

All records retrieved from the initial search were screened independently by two reviewers based on title and abstract. Full-text articles were then reviewed to confirm eligibility according to the predefined inclusion and exclusion criteria. Discrepancies between reviewers were resolved through discussion and consensus. A total of 42 cases met the inclusion criteria and were included in the final analysis.

Data Extraction

Data extraction was performed independently by two reviewers using a standardized extraction template. Extracted variables included demographic characteristics (age and sex), the timing of psychosis onset relative to COVID-19 diagnosis, and the clinical features of psychosis. Reviewers also recorded the antipsychotic medication(s) used (including dosage when reported), the use of adjunctive psychotropic or medical therapies, the time to initial clinical improvement and symptom resolution, reported adverse effects, and any relapse or recurrence during follow-up. Any discrepancies in extracted data were resolved through consensus review.

Data Synthesis

Due to heterogeneity in clinical presentations, treatment regimens, outcome reporting, and follow-up duration across case reports, quantitative synthesis and meta-analysis were not performed. Instead, findings were summarized descriptively, with emphasis on patterns of antipsychotic use, clinical response, adverse effects, and relapse rates. All frequencies and percentages were calculated as simple proportions of cases with available data for each variable. When specific data elements were not reported in individual case reports (e.g., exact antipsychotic dosage, precise time to symptom resolution), these cases were excluded from the denominator for that particular variable, and the denominator is specified for each reported percentage to ensure transparency regarding missing data. No weighted pooling, statistical adjustment, or effect size calculations were performed. Observed ranges and patterns of variation across cases are described for key variables without formal statistical measures of variability (e.g., standard deviations, confidence intervals), as such measures were not appropriate given the case report methodology, heterogeneous reporting, and small sample size.

*Limitations* The descriptive nature of this review carries several important limitations. First, the descriptive synthesis approach precludes formal statistical inference; no effect sizes, confidence intervals, or statistical significance testing were reported. Second, findings represent observed patterns in published case reports rather than population-level estimates, limiting generalizability. Third, the absence of a comparison group and the retrospective case report methodology limit causal inference regarding treatment efficacy. Fourth, heterogeneity in reporting quality, outcome definitions, and follow-up duration across case reports may affect the reliability of synthesized findings. Finally, publication bias favoring positive outcomes is a concern in case report literature.

Results

**Figure 1:**
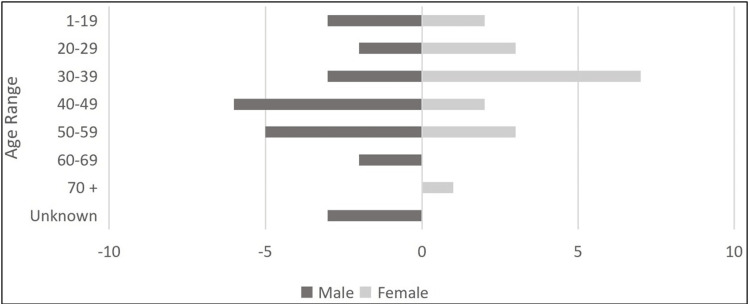
COVID-19-induced FEP distribution by sex and age. FEP, first-episode psychosis

The details of the case studies identified are listed in Table 1. Of the 42 cases, 24 (57.1%) were males and 18 (42.9%) were females who presented with FEP (Figure 1).

**Table 1:** Details of the case studies on FEP during the COVID-19 pandemic. M, male; F, female; dx, diagnosis; N/A, not available; FEP, first-episode psychosis

Study reference	Sex	Age	Antipsychotic(s) used	Dose (mg)	Additional Psychiatric medications given	Onset of Psychosis since Dx of COVID-19	Features of psychosis observed	Time until initial improvement since starting antipsychotics	Time until complete resolution since starting antipsychotics	Episodes of relapse?	Adverse side effects observed
Ahearn et al., 2023 [13]	F	34	Aripiprazole	10	Venlafaxine 150 mg	Within 3 weeks	Auditory hallucinations, hyper-religiosity, delusions, negative symptoms	N/A	N/A	Not reported	Not reported
			Olanzapine	20							
Al-Busaidi et al., 2021 [14]	M	46	Haloperidol	10	Promethazine 25 mg	2 weeks following initial admission for COVID	Auditory hallucinations, delusions	N/A	Within 2 weeks	Denied relapse	Made the patient feel "heavy and lazy."
			Olanzapine	5							
Alba et al., 2021 [15]	M	40	Aripiprazole	5	Diazepam 15 mg	2 weeks	Visual hallucinations, delusions, disorganized behavior/speech	Within 48 hours	7 days	Denied relapse	Not reported
Alihsan et al., 2023 [16]	M	40	Olanzapine	10	Valproate titrated up to 1250 mg	3 weeks	Manic symptoms	5 days	7 days	Denied relapse	Not reported
Balcioglu et al., 2023 [17]	M	58	Haloperidol	10 (IM)	Not reported	26 days	Persecutory delusions, altered behavior, pressured speech	21 days	An additional 25 days	Denied relapse	Not Reported
			Aripiprazole	5 - 20							
Bashir et al., 2022 [18]	M	16	Haloperidol	N/A	Lorazepam	3 days following discharge of the initial COVID-19 admission	Altered behavior, auditory/visual hallucinations, delusions	1 week	N/A	Denied relapse	Weight Gain
			Olanzapine	5-15							
Bakre et al., 2022 [19]	F	29	Olanzapine	10	Not reported	Immediately upon a confirmed diagnosis of her husband	Paranoid delusions, visual hallucinations, disorganized thoughts	4 days	5th day pt demonstrated considerable stability	Denied relapse	Not reported, but the family expressed concern for Olanzapine
			Aripiprazole	10							
Baral et al., 2021 [20]	M	53	Haloperidol	5 (IM)	Not reported	5 weeks	Delusions, suicidal ideation	"Next few days"	5 days following hospital discharge	Denied relapse	Not reported
Borovina et al., 2021 [21]	F	74	Risperidone	2-6	Diazepam 15 mg	1 month	Delusions, suicidal ideation with attempt	By the 10th day of hospitalization	Patient died on the 12th day of hospitalization	Not reported	Patient died on the 12th day of hospitalization
Correa-Palacio et al., 2020 [22]	M	43	Olanzapine	5	Valproic acid 500 mg/8 hours, Lorazepam 1 mg PRN	Following the COVID-19 hospitalization period	Persecutory delusions, megalomaniac beliefs	N/A	1 month after hospitalization for psychosis	Not reported	Not reported
			Paliperidone	15							
Desai et al., 2021 [23]	M	55	Haloperidol	5-10	Sodium valproate 1,000 mg, Lorazepam 2 mg,	3 weeks	Auditory hallucinations, delusions (religious in nature)	N/A	N/A	Not reported	Not reported, but change to atypical for concern of EPS
			Aripiprazole	10							
Elfil et al., 2021 [24]	F	20	Quetiapine	N/A	Lorazepam	1 month	Visual hallucinations, disorganized thoughts	N/A	N/A	Not reported	Not reported
Faisal et al., 2021 [25]	M	48	Haloperidol	N/A	Lorazepam	Since the diagnosis of COVID-19	Auditory and visual hallucinations, delusions	By the 6th day of hospitalization	13th day of hospitalization	Not reported	Not reported
			Risperidone								
Ferrando et al., 2020 - Case #1 [4]	M	30	Quetiapine	25	Not reported	Tested COVID-19 (+) when brought into ED with psychotic symptoms	Auditory hallucinations, suicidal ideation, delusions, suspiciousness	N/A	4th day of admission	Not reported	Not reported
Ferrando et al., 2020 - Case #2 [4]	F	34	Aripiprazole	2-5	Lorazepam 1 mg, Fluoxetine 10 mg, Clonazepam 0.5 mg	Tested COVID-19 (+) when brought into the ED with psychotic symptoms	Disorganized thoughts, suspicious, delusions	"Some acute improvement"	N/A	Not reported	Concern about the prolongation of the QT interval while in the ED
Gillett et al., 2020 [26]	M	37	Olanzapine	N/A	Diazepam (PRN)	Psychosis symptoms began simultaneously with COVID-19 symptoms	Delusions (religious in nature), auditory hallucinations, self-mutilation, suicidal ideation with/ attempt	"Improved within days."	N/A	Denied relapse	Not reported
			Olanzapine	N/A							
Haddad et al., 2020 [27]	M	30	Risperidone	1 x2/day	Lorazepam 1 mg x4/day, Mirtazapine 30 mg at night	1 week	Paranoid delusions, auditory hallucinations	N/A	Symptoms lasted 1 week	Denied relapse	Not reported
Huarcaya-Victoria et al., 2020 - Case #1 [28]	F	23	Ziprasidone	40	Not reported	2 days following the confirmed diagnosis of her father	Auditory hallucinations, delusions (religious in nature)	N/A	9 days	Denied relapse	Not reported
			Olanzapine	15							
Huarcaya-Victoria et al., 2020 - Case #2 [28]	F	38	Ziprasidone	20	Valproic acid 1,000 mg, Clonazepam 1 mg	14 days before admission	Auditory and visual hallucinations, disorganized thoughts, delusions (religious in nature)	N/A	10 days	Denied relapse	Not reported
			Olanzapine	20							
Huarcaya-Victoria et al., 2020 - Case #3 [28]	F	47	Haloperidol	15	Sertraline 50 mg, Valproic acid 500 mg	Tested COVID-19 (+) when brought into ED with psychotic symptoms	Auditory hallucinations, delusions of harm, suicidal ideation	N/A	10 days	Not reported	Not reported
			Quetiapine	300							
Jahan et al., 2023 [29]	F	14	Chlorpromazine	10	Not reported	2 weeks	Auditory and visual hallucinations	N/A	5 days	Not reported	Not reported
Jaworowski et al., 2020 [30]	M	N/A	Haloperidol	IM	Lorazepam	Psychosis symptoms began when the patient was admitted for COVID-19 symptoms.	Delusions (religious in nature), grandiose behavior	N/A	2 days	Not reported	Not reported
Jaworowski et al., 2020 [30]	M	N/A	Olanzapine	IM	Lorazepam	Tested COVID-19 (+) when brought into ED with psychotic symptoms	Delusions (religious in nature), grandiose behavior	N/A	2 days	Not reported	Not reported
Jaworowski et al., 2020 [30]	M	N/A	Haloperidol	IM	Lorazepam	3 days following admission for COVID-19 symptoms	Delusions (religious in nature), grandiose behavior, paranoia	N/A	2 days	Not reported	Not reported
Kazi et al., 2021 [31]	F	49	Aripiprazole	5-7	Lorazepam 2 mg, Escitalopram 10-20 mg, Mirtazapine 15 mg	Tested COVID-19 (+) when brought into ED with psychotic symptoms	Paranoid delusions, constricted affect, suicidal ideation	Improvement by day 4 to day 15 and again from day 17 to day 21	N/A	After 8 months of initial hospitalization	Observed akathisia 2 months into taking Olanzapine
			Haloperidol	Single dose							
			Olanzapine	7.5-25							
			Aripiprazole	2							
			Discharged w/: Olanzapine	25							
Kazi et al., 2021 [31]	F	56	Aripiprazole	5	Not reported	Psychosis symptoms began a few days before admission for COVID-19 symptoms.	Auditory and visual hallucinations, delusions, agitated behavior	N/A	N/A	No follow-up reported after discharge	Not reported
			Olanzapine	5 (IM)							
Lim et al., 2020 [32]	F	55	Haloperidol	0.5 BID	Not reported	Symptoms began 1 day following confirmed COVID-19 dx	Visual hallucinations, paranoid delusions	By 20th day of case (6th day on Risperidone)	By the 52nd day of the case	Not reported	Not reported
			Risperidone	0.5							
Lorenzo-Villalba et al., 2020 [33]	F	33	Olanzapine	10	Not reported	Tested COVID-19 (+) when brought into ED with psychotic symptoms	Auditory hallucinations, altered behavior, incoherent speech	N/A	Day 14 of hospital admission	Not reported	Not reported
Majadas et al., 2020 [34]	M	63	While admitted: Risperidone	2.5	Not reported	Psychosis symptoms began when the patient was admitted for COVID-19 symptoms	Delusions, incoherent thought, and speech	"Improved in parallel with the respiratory disorder."	N/A	Readmitted for delusions + auditory hallucinations 7 days following initial discharge	Not reported
			Discharged w/: Risperidone	2							
			2nd Admission: Risperidone	6							
Meeder et al., 2022 - Case #2 [35]	M	17	Haloperidol	As needed	Lithium 300 mg BID for mania, Lorazepam as needed, Diphenhydramine as needed	Tested COVID-19 (+) when brought into the ED with psychotic symptoms	Delusions, disorganized speech, and thought	"Patient was discharged 10 days later."	N/A	Not reported	Not reported
			Olanzapine	N/A							
			Discharged w/Olanzapine	7.5 (BID)							
Marinova et al., 2023 [36]	M	25	Zuclopenthixol	100	Promethazine 50 mg IM/d - 100 mg IM/d, Valproate 1,000 mg IV/d -- 1,500 mg/d, Biperiden 4 mg/d	Tested COVID-19 (+) 25 days before admission for symptoms	Auditory hallucinations, delusions, disorganized thought, and aggressive behavior	N/A	N/A	Not reported	While on Zuclopenthixol, lab findings showed leukocytosis, lymphopenia, and eosinopenia.
			Haloperidol	10							
			Risperidone	6							
Mirza et al., 2020 [37]	M	53	Olanzapine	5	Not reported	Showed COVID-19 features when brought into the ED for an attempt at suicide	Suicidal ideation + attempt, auditory hallucinations	N/A	N/A	Not reported	Not reported
Noone et al., 2020 [38]	M	49	Haloperidol	2	Not reported	Diagnosed with presumed COVID-19 (+) 3 weeks before admission for symptoms	Auditory hallucinations, delusions of grandiosity	Improved over 2.5 weeks	N/A	Not reported	Not reported
			Olanzapine	2.5							
			Switched to: Quetiapine	Up to 150							
Noone et al., 2020 [38]	F	34	Risperidone	1 (BID)	Not reported	Tested COVID-19 (+) when brought into ED when first screened for severe agitation and anxiety 2.5 weeks ago	"Bizarre behavior," persecutory delusions	Showed significant improvement 1 week following inpatient psychiatry	N/A	Not reported	Not reported
Parker et al., 2021 [39]	M	57	Haloperidol	5	Lorazepam 2 mg	Tested COVID-19 (+) when brought into ED with psychotic symptoms	Delusions, hallucinations, disorganized thought, and behavior	N/A	N/A	N/A	Observed to show a prolonged QTc Interval of 490 ms with Haloperidol
			Aripiprazole	5							
Puiu et al., 2023 [40]	M	28	Haloperidol	5 (BID)	Diazepam 10 mg IM BID, Divalproex Sodium 1,500 mg oral	1 week following discharge for COVID-19 symptoms	Auditory and visual hallucinations, delusions, and aggressive behavior	N/A	"Treatment with full resolution"	Denied relapse	Not reported
			Aripiprazole	15							
Santos, 2021 [41]	M	61	Risperidone	3-5	Lorazepam 0.5 mg x 3 days	1-month presentation to ED, 2 days before COVID-19 diagnosis	Delusions of jealousy, auditory hallucinations	15 days	3 months - Risperidone 1 mg	Not reported	Not reported
Sen, 2021 [42]	F	33	Haloperidol	20	Biperiden 10 mg/d, Olanzapine 20 mg/d	COVID-19 antibodies detected, no formal dx	Delusions, acute mania/BPD	N/A	N/A	N/A	N/A
Smith et al., 2020 [43]	F	36	Olanzapine	5	Clonazepam 0.5 mg twice a day	4 days	Delusions, decreased sleep	7 days	N/A	N/A	N/A
			Risperidone	3							
Thomas et al., 2022 [44]	F	15	Olanzapine	7.5 (5 at bedtime, 2.5 added in the AM)	Haloperidol for agitation	2.5 weeks	Paranoia, delusions, hallucinations	5 days	1 week	N/A	N/A
Tuna et al., 2020 [45]	F	52	Haloperidol	10 mg/d	Biperiden 5 mg/d parenterally	Tested positive on admission	Auditory hallucinations, SI, delusions	1 week	N/A	N/A	N/A
Valikhani et al., 2023 [46]	M	16	Quetiapine	25	Valproate 200 mg/d Clonazepam 1 mg 1 hour before bed	5 days	Hallucinations, insomnia, delusions	15 days	40 days	N/A	N/A
			Risperidone	2							

Among the 42 case reports, psychotic features were most frequently observed in patients aged 30-39 years with 10 (23.8%) cases, followed by equal distributions in the 40-49 and 50-59 age groups at 8 (19.0%) cases each. Younger patients aged 1-19 and 20-29 years also showed equal distributions, with 5 cases (11.9%) each. The remaining cases included 2 (4.8%) patients aged 60-69 years, 1 (2.4%) patient aged 70 years or above, and 3 (7.1%) cases with unspecified age (Figure 1).

Out of the 42 identified cases, 21 involved monotherapy with a single antipsychotic. Olanzapine was the most frequently used agent in six cases (28.6%), followed by haloperidol in five cases (23.8%) and risperidone in five cases (23.8%). Aripiprazole and quetiapine were each used in two cases (9.5%), while chlorpromazine was used in one case (4.8%) (Table 2). Multi-antipsychotic management was employed in the remaining 21 cases, with the haloperidol-aripiprazole combination being most common in four cases (19.0%). Other combinations included haloperidol-olanzapine in three cases (14.3%), haloperidol-risperidone in two cases (9.5%), and aripiprazole-olanzapine in two cases (9.5%) (Table 2).

**Table 2:** Antipsychotic management by use category.

Usage frequency	Antipsychotic management	Total # of cases	% within group	Group total
Single-Use	Aripiprazole	2	9.5	21
Single-Use	Chlorpromazine	1	4.8	21
Single-Use	Haloperidol	5	23.8	21
Single-Use	Olanzapine	6	28.6	21
Single-Use	Quetiapine	2	9.5	21
Single-Use	Risperidone	5	23.8	21
Multi-Use	(1.) Aripiprazole → (2.) Olanzapine	2	9.5	21
Multi-Use	(1.) Haloperidol → (2.) Olanzapine	3	14.3	21
Multi-Use	(1.) Haloperidol → (2.) Aripiprazole	4	19.0	21
Multi-Use	(1.) Haloperidol → (2.) Risperidone	2	9.5	21
Multi-Use	(1.) Haloperidol → (2.) Quetiapine	1	4.8	21
Multi-Use	(1.) Haloperidol + Olanzapine → (2.) Quetiapine	1	4.8	21
Multi-Use	(1.) Haloperidol + Aripiprazole → (2.) Olanzapine + Aripiprazole → (3.) Olanzapine	1	4.8	21
Multi-Use	(1.) Olanzapine → (2.) Aripiprazole	1	4.8	21
Multi-Use	(1.) Olanzapine + Paliperidone	1	4.8	21
Multi-Use	(1.) Olanzapine → (2.) Risperidone	1	4.8	21
Multi-Use	(1.) Quetiapine → (2.) Risperidone	1	4.8	21
Multi-Use	(1.) Ziprasidone → (2.) Olanzapine	2	9.5	21
Multi-Use	(1.) Zuclopenthixol → (2.) Haloperidol → (3.) Risperidone	1	4.8	21

Out of the 42 cases, six (14.3%) used only typical antipsychotics, and 23 (54.8%) used only atypical antipsychotics. A typical antipsychotic (haloperidol only) was used in combination with an atypical antipsychotic in 12 cases (28.6%). In contrast, only one case (2.4%) used a typical antipsychotic other than haloperidol in combination with an atypical antipsychotic to treat psychotic symptoms (Table 3).

**Table 3:** COVID-19-induced FEP distribution by typical versus atypical antipsychotic use, with and without haloperidol combination. FEP, first-episode psychosis

Treatment type	*n* (%) (42 cases)
Typical only	6 (14.3%)
Atypical only	23 (54.8%)
Typical (Haloperidol only) + atypical in combination	12 (28.6%)
Typical (not Haloperidol) + atypical in combination	1 (2.4%)

Out of 42 cases, a total of nine different antipsychotics were used at various times during patient management. The most frequently used antipsychotics were haloperidol and olanzapine, each in 18 cases (42.9%); aripiprazole and risperidone, each in 10 cases (23.8%); quetiapine in five cases (11.9%); ziprasidone in two cases (4.8%); and chlorpromazine, paliperidone, and zuclopenthixol in one case each (2.4%) (Table 4).

**Table 4:** Antipsychotics used, frequency, range of dosages, and average dose administered across all cases. Note: Percentages were calculated based on the total sample of 42 cases. Percentages sum to >100% because multiple antipsychotics were used in some cases.

Antipsychotic frequency of use in total cases	Dose range presented in total cases (per day)	Average dose administered (per day)	Most frequent dose used (per day)
Aripiprazole (10, 23.8%)	2-20 mg	7.4 mg	5 mg
Chlorpromazine (1, 2.4%)	10 mg	10 mg	10 mg
Haloperidol (18, 42.9%)	1-20 mg	6.1 mg	10 mg
Olanzapine (18, 42.9%)	2.5-25 mg	10.9 mg	5 mg
Paliperidone (1, 2.4%)	15 mg	15 mg	15 mg
Quetiapine (5, 11.9%)	25-300 mg	131.3 mg	25, 50, 150, and 300 mg
Risperidone 10 (23.8%)	0.5-6 mg	3.0 mg	2 mg
Ziprasidone (2, 4.8%)	20-40 mg	30 mg	20 and 40 mg
Zuclopenthixol (1, 2.4%)	100 mg	100 mg	100 mg

Among the total cases that administered antipsychotics, side effects were reported in five cases (11.9%), zero cases had denied any observed side effects, and 37 cases (88.1%) failed to report or mention any side effects exhibited by the patient. The reported five cases of side effects included fatigue, weight gain, akathisia, abnormal CBC levels (leukocytosis), and prolonged QT interval.

Among the total cases treated with antipsychotics with complete resolution of psychotic symptoms, relapses of psychotic symptoms were observed in two (4.8%) patients, 11 cases (26.2%) denied any relapse through follow-up, and 29 cases (69.0%) did not report any relapse.

## Review

This report identifies 42 cases of new-onset psychosis occurring at the time or following a COVID-19 diagnosis, with no prior psychiatric history. It provides a summary of demographic characteristics and the course of illness (including relapses) and primarily focuses on the management of various antipsychotic strategies. While the cases exhibit a gender distribution of 24 (57.1%) males and 18 (42.9%) females, this study does not consider gender as a contributing factor in the management style due to the limited sample size. Previous studies have similarly shown no association between gender and COVID-19 psychosis [47].

The initial antipsychotics, referred to now as first-generation or typical antipsychotics, chlorpromazine, haloperidol, and fluphenazine, were the pioneering medications in the field of antipsychotics [48]. First-generation or typical antipsychotics act as dopamine receptor antagonists, blocking the D2 receptors of dopaminergic neurotransmission and blocking noradrenergic, cholinergic, and histaminergic pathways [49]. First-generation antipsychotics have an array of adverse effects, especially their extrapyramidal side effects. Typical antipsychotics may be unfavorable due to their anticholinergic side effects, such as dry mouth, constipation, and urinary retention, and their histamine-blocking effects, including sedation [50]. They have the potential to lower the seizure threshold, making patients with a history of seizures more susceptible, while prolonging the QTc interval with delayed atrial, ventricular contraction, and other cardiac conduction abnormalities that may be seen [49]. Leukopenia, blood dyscrasia, and thrombocytopenia are uncommon side effects. Increased serum prolactin, galactorrhea, breast enlargement, amenorrhea, impotence in men, and anorgasmia in women can be attributed to the blocking of the dopaminergic impact [49]. Specifically, typical antipsychotics have their unique side effects. Chlorpromazine is associated with jaundice, allergic dermatitis, photosensitivity, blue-gray skin discoloration, and benign pigmentation of the lens and cornea [49,51].

Second-generation antipsychotics or atypical antipsychotics act as serotonin-dopamine antagonists. Atypical antipsychotics act by blocking D2 dopamine and serotonin receptors, most commonly 5-HT2A [52]. Atypical antipsychotics are known for decreased risk of extrapyramidal side effects compared to typical antipsychotics, believed to be due to a reduced binding affinity for dopamine receptors, while associated with weight gain and metabolic syndrome [48.49]. Risperidone was used in ten reported cases and is associated with anxiety, sedation, dizziness, and extrapyramidal symptoms [49]. Paliperidone, used in one instance, is associated with temperature sensitivity and QTc prolongation [49]. Olanzapine was used in 18 reported cases, with a side-effect profile including increased appetite, somnolence, and weight gain [49]. Quetiapine (five cases) is associated with drowsiness, dizziness, and hypotension. Clozapine can cause hypersalivation, tachycardia, hypotension, anticholinergic side effects, suppression of dyskinesia, agranulocytosis, cardiomyopathy, and myocarditis [49]. Aripiprazole (10 cases) is associated with agitation, headache, and akathisia-like restlessness. Ziprasidone (two cases) is associated with minimal weight gain but prolonged QTc. Additionally, ziprasidone is linked to the lowest lifestyle costs among second-generation antipsychotics due to their higher avoidance rates of cardiovascular events and diabetes [53]. Patients diagnosed with psychosis often exhibit low adherence rates to antipsychotic medications, as evidenced by a review of 38 studies with 51,796 patients showing a mean adherence rate of only 42% in schizophrenia [54].

Atypical antipsychotics were more commonly used than typical antipsychotics in treating COVID-19 FEP, notably when excluding haloperidol for initial acute management. Atypical antipsychotics are preferred due to their lower likelihood of causing extrapyramidal and endocrine adverse symptoms, promoting better patient compliance [55].

Our data indicate frequent use of haloperidol (18 cases) in the early stages of management and Olanzapine (18 cases) in the later stages. Although widely prescribed for agitation in psychotic disorders and acute mania, haloperidol has multiple FDA-approved uses, including schizophrenia, Tourette’s syndrome, severe behavioral disorders, and hyperactivity. It is also used off-label for chemotherapy-induced nausea, vomiting, and hiccups [56]. However, intravenous use of haloperidol poses risks such as abnormal heart rhythm, ventricular arrhythmia, torsades de pointes, and potentially fatal outcomes [49]. Despite these concerns, haloperidol remains the primary choice for initial management in acute settings.

For both atypical and typical antipsychotics, there are various methods of delivery, including oral, parenteral, extended-release, or long-acting injectable [49]. However, limited information was provided within the studies regarding route administration during admissions and hospitalization to document accurately. 

It is important to note that potential confounders may contribute to FEP. For instance, the use of steroids for the treatment of COVID-19 pneumonia may play a critical role in the development of FEP [57]. Additionally, with prolonged immobilization with ventilation, the use of steroids, and cytokine storm with immune and inflammatory changes occurring, the potential of neuropsychiatric symptoms increases [7,58-59]. For instance, elevated C-reactive protein (CRP) as a potential peripheral marker for immune activation may play a role in schizophrenia and related psychotic features and psychosis [60]. However, with proinflammatory cytokines interleukin-6 (IL-6), tumor necrosis factor-alpha (TNF-alpha), IL-8, IL-10, IL-2R, and CRP being all elevated in patients with COVID-19, can reflect disease severity [61]. Furthermore, measurement of a broader array of cytokines in the peripheral blood and cerebrospinal fluid (via lumbar puncture) would better characterize immune activation peripherally. Measurement of COVID-19 RNA in cerebrospinal fluid could indicate virological invasion into the CNS.

The potential of immune-modulation therapies, such as IL-6 inhibitor agents and melatonin, is under investigation for COVID-19 [62,63]. Other therapies, such as intravenous immunoglobulin, cytokine-blocking medications, and Janus kinase (JAK) inhibitors, may show promising results in parallel with antipsychotics.

Due to the small sample size, the study doesn't allow conclusive statements about differences in onset rates, therapy effectiveness, or complete resolution among the administered antipsychotics. The limited sample size does not allow for definitive conclusions about underlying risk factors or predictive patterns. The absence of neuroimaging in confirmed COVID-19 cases does not exclude potential CNS pathology. More sensitive imaging techniques could enhance understanding.

Consequently, no specific protocol regarding antipsychotic preferences can be established for the management of FEP in COVID-19 cases. However, the collected data highlight promising results, aiding therapeutic decision-making in rare cases of COVID-19-induced FEP in clinical settings.

## Conclusions

Psychosis, a complex condition with various causes, is increasingly seen in patients with or after a COVID-19 infection. Ongoing research on SARS-CoV-2 and its neuroinvasive mechanisms is necessary for understanding the pathophysiology of COVID-19-induced FEP. This review of 42 case studies demonstrates that antipsychotic medications are effective in managing psychotic symptoms in patients with no prior psychiatric history who develop new-onset psychosis during or after COVID-19 infection. These findings show that atypical antipsychotics were preferred in many cases, with both olanzapine and haloperidol being the most frequently used medications, each used in 18 cases throughout the duration of treatment. While atypical antipsychotics show benefit in terms of decreased extrapyramidal side effects, Haloperidol remained commonly used for acute management despite its association with such symptoms. The demographic analysis showed a higher incidence among males than females, with the 30-39 age group representing the most affected population. Treatment outcomes were overall favorable, with many patients achieving improvement or resolution of psychotic symptoms after antipsychotic treatment. Side effects were reported in only a small number of cases - 37 reported no adverse effects - making it difficult to draw comprehensive conclusions about tolerability. Relapses of psychotic symptoms were observed in only two patients, though an increased number of cases did not report follow-up documentation regarding relapse status. Important considerations should be given regarding potential confounders such as the use of corticosteroids for COVID-19 treatment, immune dysregulation, cytokine storm, and the direct neurotropic effects of the virus. As the understanding of COVID-19's neuropsychiatric sequelae continues to grow, clinicians should maintain vigilance for psychotic symptoms in COVID-19 patients, recognize the importance of timely diagnosis and treatment, and consider the broader implications for long-term neuropsychiatric care in post-COVID populations. Further research involving larger sample sizes and more controlled studies is necessary to establish standardized treatment protocols, identify risk stratification criteria, and optimize therapeutic strategies for this emerging clinical phenomenon.
